# Different Roles of Heat Shock Proteins (70 kDa) During Abiotic Stresses in Barley (*Hordeum vulgare*) Genotypes

**DOI:** 10.3390/plants8080248

**Published:** 2019-07-26

**Authors:** Simone Landi, Giorgia Capasso, Fatma Ezzahra Ben Azaiez, Salma Jallouli, Sawsen Ayadi, Youssef Trifa, Sergio Esposito

**Affiliations:** 1 Dipartimento di Biologia, Università di Napoli ‘‘Federico II’’, Via Cinthia, I-80126 Napoli, Italy; 2 Department of Agronomy and Plant Biotechnology, National Institute of Agronomy, 1082 Tunis, Tunisia

**Keywords:** drought, salinity, poaceae, HSP70, landraces, mediterranean area, chaperons, abiotic stress

## Abstract

In this work, the involvement of heat shock proteins (HSP70) in barley (*Hordeum vulgare*) has been studied in response to drought and salinity. Thus, 3 barley genotypes usually cultivated and/or selected in Italy, 3 Middle East/North Africa landraces and genotypes and 1 improved genotype from ICARDA have been studied to identify those varieties showing the best stress response. Preliminarily, a bioinformatic characterization of the HSP70s protein family in barley has been made by using annotated Arabidopsis protein sequences. This study identified 20 putative HSP70s orthologs in the barley genome. The construction of un-rooted phylogenetic trees showed the partition into four main branches, and multiple subcellular localizations. The enhanced HSP70s presence upon salt and drought stress was investigated by both immunoblotting and expression analyses. It is worth noting the Northern Africa landraces showed peculiar tolerance behavior versus drought and salt stresses. The drought and salinity conditions indicated the involvement of specific HSP70s to counteract abiotic stress. Particularly, the expression of cytosolic MLOC_67581, mitochondrial MLOC_50972, and encoding for HSP70 isoforms showed different expressions and occurrence upon stress. Therefore, genotypes originated in the semi-arid area of the Mediterranean area can represent an important genetic source for the improvement of commonly cultivated high-yielding varieties.

## 1. Introduction

The Heat Shock Proteins 70 (HSP70s) are a subfamily of the heat shock proteins, a well-known class of molecular chaperons involved in an abiotic stress response [1]. The HSP70s present a nucleotide binding domain (NBD) of 45kDa showing ATPase activity and a 15 kDa substrate binding domain (SBD) with a C-terminal domain covering the SBD [2]. The C-term region acts as a lid and cooperate with SBD in substrates binding [3,4]. The SBD differs among the species and usually presents organelle specific motifs [5,6]. Particularly, a plants’ HSP70s show several different subcellular localizations, namely cytosolic, nuclear, endoplasmic reticulum, chloroplastic and mitochondrial [7,8]. 

The HSP70s play a central role in the stabilization of proteins both under optimal conditions and during stress, thus helping cellular machinery in verifying protein quality and regulating protein degradation [9]. Particularly, the HSP70s avoid the aggregation of polypeptides and facilitate the proteins’ maturation [10]. During abiotic stresses, the HSP70s act on misfolded and truncated proteins thus protecting the cells and the tissues [11,12]. This mechanism is regulated by heat shock factors (Hsfs), a group of transcription factors regulating HSP70s expression [12,13]. 

The HSP70 activation during environmental perturbations has been reported in different plants such as *Arabidopsis thaliana, Brachypodium distachyon, Glycine max, Capsicuum annum, Solanum lycopersium* and others [4,11,12,13,14]. Particularly, water scarcity and soil salinity, together with nitrogen deprivation, represent critical factors for a crops’ production [15,16,17,18]. 

Nowadays, the improvement of crop yields in adverse environments represents one of the most impelling topics [19,20] (Tester and Langridge, 2010; Cobb et al., 2013). Particularly, the key role of Poaceae in food demand is well recognized: Rice (*Oryza sativa*), wheat (*Triticuum aestivum*), maize (*Zea mays*) and barley (*Hordeum vulgare*) represent the most important food sources for the majority of the world’s population [21,22,23,24]. In this context, barley represents a critical agronomic resource in semi-desert environments, especially in the Southern Europe and Northern Africa. In developing countries, barley is a critical component of cereal rotations, playing a key role in the integrated crop-livestock production systems. It provides a stable source for sustaining smallholder farmers, replacing wheat or other cereals in many arid areas [25]. Barley shows a natural resistance to exogenous stimuli, thus representing the most tolerant Poacea against abiotic stresses [23]. Furthermore, 40% of alleles were maintained in cultivated barley compared with the historic progenitor (*Hordeum spontaneum*—[26]). This wild ancestor showed remarkable tolerance to salt, drought and heavy metals stress, but domestication by humans and, more recently, breeding programs produced high-yielding barley cultivars that are, on the other hand, more sensitive to abiotic stress, making this aspect a critical issue in barley as well [23,24,27]. 

Therefore, the ancestral and local cereal landraces that originated from saline and emarginated environments could represent a source of genetic diversity [25,28,29]. Therefore, the breeding research focus is moved to minimizing the gap between yields under optimal and stress conditions [25,30], contributing to the adaptation on, and contrast to, climate change [31]. 

To the authors’ knowledge, no molecular and enzymatic studies are present about the HSP70s in barley landraces from the Mediterranean area. The goal of this research is the evaluation of the specific presence of the HSP70s isoforms under salt and drought stress in different barley genotypes. Therefore, a bioinformatic characterization of the HSP70s protein family in barley has been made, and seven barley genotypes and landraces obtained from Italy and Northern Africa were used to investigate the occurrence of the HSP70s under abiotic stress conditions.

## 2. Results

### 2.1. HSP70s Showed Peculiar Roles against Abiotic Stress in Barley

#### 2.1.1. NaCl and PEG Effects on Barley Plants

**Figure 1:**
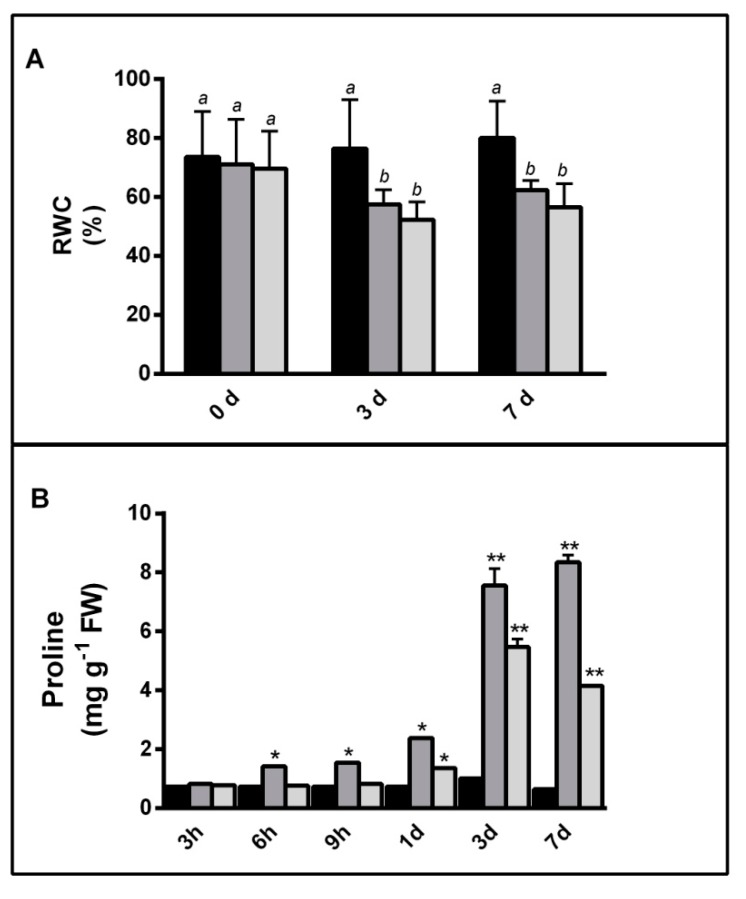
(**A**) The relative water content (RWC) and (**B**) Proline content, in leaves of barley (*Hordeum vulgare* cv. Nure) grown under controlled conditions (black bars); salt stress (150 mM NaCl—medium grey bars) and drought (10% PEG—light grey bars) at given times. In (**A**) statistically similar data are grouped by letters a and b in the control, salt stress and drought groups, respectively. In (**B**) asterisks indicate a significance between stressed and the control plants. * = *p* < 0.05; ** = *p* < 0.001.

In order to characterize the HSP70s role(s) upon salt and drought stress, this study selected the commercial variety *Hordeum vulgare* Nure. To describe a general response pattern of barley plants to salinity and water deficit, short-term severe stress conditions (10% PEG and 150 mM NaCl) were imposed to plants grown in hydroponics. The stress response was monitored using relative water content (RWC) and proline content. As described in Figure 1A, after 3 days of treatments, the barley plants showed the maximum stress effects. Particularly, a significant 21% and 24% decreased in RWC was reported after 3 days of treatments and remained stable up to 7 days. Furthermore, the proline content increased from approximately 2 to 6–7-fold change, in NaCl stressed plants. Intriguingly, the drought induced a higher proline from 3 to 12-fold change within 7d (Figure 1B). 

#### 2.1.2. Barley HSP70 Isoforms Showed Specific Occurrence upon Abiotic Stresses

The HSP70s roles upon salt and drought stress from Nure were investigated using the different occurrence of isoforms together with specific gene expression analyses. 

**Figure 2:**
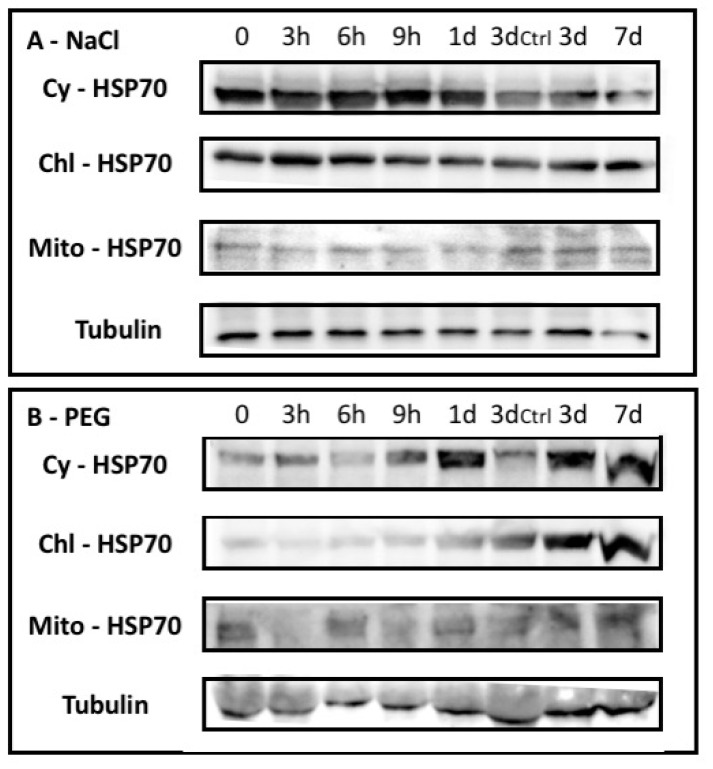
The immunoblotting of leaf extracts from Barley Nure plants exposed to (**A**) salt stress (NaCl); and (**B**) Drought (PEG) collected at given times. In the lane 3ctrl extracts from untreated plants after 3d were loaded. Immunoblotting was performed by using antibodies raised abainst cytosolic (Cy-HSP70); chloroplastic (Chl-HSP70) and mitochondrial (Mito-HSP70). The control blots using anti-tubulin antisera are shown.

A western blotting approach using cyt-, chl-, and mito-HSP70 antibodies showed peculiar behavior for the different HSP70 isoforms upon abiotic stresses (Figure 2). The Salinity induced a slight increase of cytosolic HSP70 occurrence after 3 h. On the contrary, chloroplastic HSP70 remained substantially unchanged in both the control and stressed plants. Mitochondrial HSP70 was barely detectable and slightly increased after 1 day of treatment. 

The drought stress showed an increase of all HSP70 isoforms after 3 days of treatment, but cytosolic HSP70 increased soon after 9 h. 

### 2.2. HSP70s in Barley: A Bioinformatic Overview

In order to investigate the role(s) of different HSP70s upon abiotic stress in crops, this study performed an extensive bioinformatic approach to characterize this gene family in *Hordeum vulgare*. Using annotated *Arabidopsis thaliana* protein sequences (at https://www.arabidopsis.org), the authors identified putative HSP70s orthologs in barley genome at https://ics.hutton.ac.uk/morexGenes/ and http://plants.ensembl.org/index.html, showing a total of 20 HSP70s genes (Table 1).

The intracellular localization of the HSP70 obtained by the phylogenetic analysis was confirmed by the online software Prot Comp9.0 server4 (http://linux1.softberry.com/berry.phtml), mitoproth server (http//ihg.gsf.de/ihg/mitoprot.html) and using Chloro P software (http://www.cbs.dtu.dk/services/ChloroP/)—(Table 1). Furthermore, in order to characterize the identified proteins, the pfam database was used—each protein, with the exception of MLOC_55096, retrieved the HSP70s pfam domain (PF00012.20). The prokaryotic domain PF06723.13 was retrieved in 17 protein as well. This singularity was interpreted by considering PF00012.20 and PF06723.13 belonging to the same pfam clan (CL0108).

**Figure 3:**
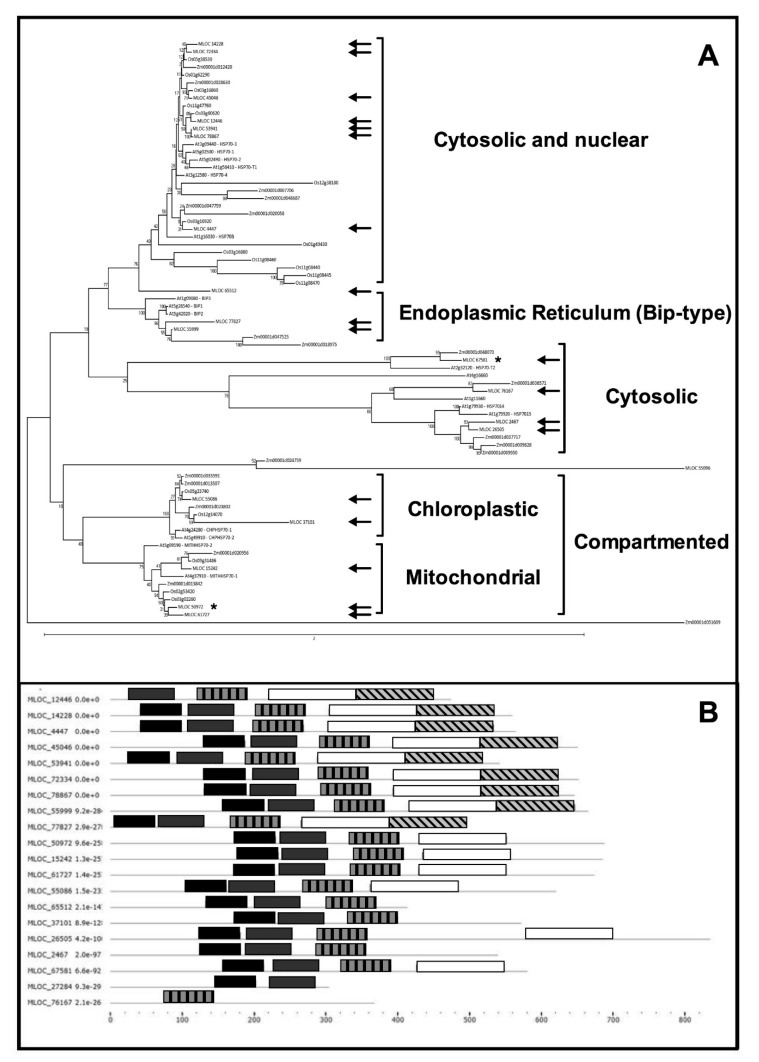
(**A**) A phylogenetic tree obtained by comparison of barley HSP70 amino acidic sequences of translated genes performed versus the correspondent *Arabidopsis thaliana*, *Oryza sativa* and *Zea mays* sequences. The predicted subcellular localizations are indicated for the four main branches; the arrows indicate the position of barley HSP70 isoforms. The asterisks indicate the two barley isoforms utilized for further expression studies. See text for further details. (**B**) Conserved domain analysis of barley HSP70 proteins, obtained using the MEME bioinformatic tools (http://meme-suite.org). The different color boxes represent different types of domains: ATPase binding domains (black, grey and vertical line pattern), substrate binding domain (white block) and the c-term lid (crossing line pattern). The number indicated the position of amino acids in protein sequences.

With the aim of identifying the different HSP70 sub-families and their phylogenetic connections, a comparison of putative protein sequences was made versus *Arabidopsis thaliana* and Poaceae (Rice—*Oryza sativa* and Mais—*Zea mays*) sequences, thus obtaining an un-rooted phylogenetic tree (Figure 3a). This showed the partition into four main branches. Group 1 includes cytosolic/nuclear HSP70s encoded by MLOC_14228, MLOC_72334 MLOC_45046, MLOC_12446, MLOC_53941, MLOC_78867 and MLOC_4447. This group of proteins showed an interesting similarity with HSP70 1-2-3-4-T1 and HSP70B from *Arabidopsis thaliana*. A second group includes the HSP70s localized within the endoplasmatic reticulum, generally recognized as BIP proteins. These HSP70s present an ATP-binding domain at the N-terminal and a C-terminal domain binding targets by recognition of the hydrophobic patches typical of improperly/incompletely folded proteins. Group 2 includes the HSP70s encoded by MLOC_77827 and MLOC_55999. 

A third group includes cytoplasmic HvHSP70s similar to *Arabidopsis thaliana* HSP70-T2 (MLOC_67581) and *At*HSP7014-15 (MLOC_2467, MLOC_26505). 

Further, the fourth branch identified the HSP70s present into organelles. In fact, this splits in two further forks, including chloroplastic-HSP70s (MLOC_55086 and MLOC_37101) and mitochondrial-HSP70s (MLOC_15242, MLOC_50972 and MLOC_61727). Finally, two HSP70s apparently cluster outside the major branches (MLOC_65512 and MLOC_55096). 

A conserved domain analysis, using the MEME bioinformatic tools (http://meme-suite.org), was carried out to investigate the HSP70s protein structures. As showed in Figure 3B, MLOC_55096 protein showed no-HSP70 domains and probably do not represent a member of this class of proteins, therefore it was excluded in this analysis. MLOC_65512, MLOC_37101, MLOC_2467, MLOC_26505 and MLOC_76167 showed a less conserved substrate binding domain (Figure 3B). 

Furthermore, compartmented HSP70s MLOC_50972, MLOC_15242, MLOC_61727, MLOC_55086 and MLOC_37101 showed no lid domain. This property is common to other cytosolic HSP70s, such as MLOC_65512, MLOC_2467, MLOC_67581 (Figure 3B).

A bioinformatic survey on *Arabidopsis* orthologous was performed to identify the best co-expressed genes (Supplemental Table S1). This verifies the cross-interaction among various HSP70s and/or other members of the heat shock proteins family (HSP20s, HSP80s). It is also worth noting the interesting relationship showed by the cytosolic HSP70 1-2-3 (*At*5g02490, *At*5g02500, *At*3g09440), and a mitochondrial HSP70 (*At*5g09590) which showed strictly a co-expression. These three cytosolic HSP70s were suggested to be participating to the abiotic stress response (Leng et al., 2017). These HSP70s showed a co-expression with stress related genes as glutathione-s-transferase (*At*5g42150), pyrroline-5-carboxylase-reductase (*At*5g14800), FTSH proteases 4 and 10 (*At*2g26140 and *At*1g07510), ascorbate peroxidase and dehydroascorbate reductase (*At*1g07890 and *At*1g75270) and others (Data not shown). 

Furthermore, an expression analysis of barley HSP70s was attained by using the online RNA-seq dataset at https://ics.hutton.ac.uk/morexGenes/. As showed in Table 2, 10 HSP70s genes (MLOC_2467, MLOC_12446, MLOC_26505, MLOC_37101, MLOC_50972, MLOC_53941, MLOC_55086, MLOC_55999, MLOC_76167, MLOC_78867) are constitutively expressed in each tissue/development stage. Among these, MLOC_12446 appears the barley HSP70 predominant expressed gene. Furthermore, 4 HSP70 genes (MLOC_14228, MLOC_4447, MLOC_45046 and MLOC_67581) showed seedling and/or grains specific expression, while 4 HSP70s genes (MLOC_55096, MLOC_65512 and MLOC_77827) showed no FPKM counts in the control conditions, and these are probably regulated upon specific stimuli. 

### 2.3. Analysis of cis-Acting Elements in the HSP70s Promoters

To investigate the regulation patterns of HSP70s, a search on the cis-elements in the promoter regions (1500 bp upstream from to the start codons) was made by using the PLANTCARE database (Table 3). Particularly, the barley HSP70 genes highlighted different behaviors to counteract the abiotic stresses. The drought sensitive elements (MBS) were found in MLOC_12446, MLOC_2467, MLOC_45046, MLOC_50972, MLOC_53941, MLOC_55096, MLOC_65512, MLOC_67581, MLOC_72334 and MLOC_78867; heat-responsive elements (HSE) were found in MLOC_12446, MLOC_2467, MLOC_26505, MLOC_4447, MLOC_45046, MLOC_50972, MLOC_55096, MLOC_55999, MLOC_61727, MLOC_65512 and MLOC_78867. 

In addition, TC-rich repeats motif (cis-acting element related to defense and stress response) were identified in MLOC_12446, MLOC_14228, MLOC_2467, MLOC_26505, MLOC_4447, MLOC_50972, MLOC_53941, MLOC_55999, MLOC_65512 and MLOC_72334. 

Furthermore, the HSP70s genes from barley exhibiting different patterns of cis acting elements in response to plant phytoregulators, such as abscissic Acid (ABRE elements and IIB motif), Gibberelic Acid (GARE elements and Box P), Auxin (TGA elements), Ethylene (ERE elements) and Methyl Jasmonate (CGTCA and TGAGC motifs).

Interestingly, MLOC_67581 presents 4 ABRE elements (responsive to ABA), the highest number among all barley HSP70s, suggesting an effective abiotic stress induction of this cytosolic isoform. On the opposite, MLOC_12446, MLOC_2467, MLOC_4447, MLOC_50972, MLOC_61727, MLOC_65512, MLOC_72334 present no ABRE elements. Among the latter group (no-ABRE elements), MLOC_50972 interestingly shows 3 BOX W1 (fungal elicitor), and 3 CGTCA elements (Me-Jasmonate responsiveness) indicating this mitochondrial isoform as strictly specific versus a pathogen attack. It should be noted that also MLOC_72334 (3 BOX W1, 2 CGTCA elements), and MLOC_61727 (1 BOX W1, 4 CGTCA elements) are suspected to be highly sensitive to fungi/pathogens.

### 2.4. Real Time PCR of Selected HSP70 Isoforms 

The bioinformatic analysis of promoter regions allowed the identification of at least two HSP70 isoforms that appear differently regulated upon stress. As previously described, MLOC_67581 encodes for a cytosolic isoform that is characterized by the highest presence of ABA responsive elements (4 ABRE). On the other hand, the biotic stress related elements are strongly limited in the promoter of this gene. 

In contrast, the mitochondrial MLOC_50972 does not present ABRE (and ARE) elements, but this promoter region shows the highest number of elements responsive to biotic stress: 3 Box W1, 3 CGTA (MeJA responsive) and 1 TC-rich elements, thus suggesting that this HSP70 isoform is induced under fungal/pathogen attack, but scarcely reactive to abiotic stress. 

Therefore, this study performed a qRT-PCR expression analysis of these two genes to investigate their possible different expression rates upon abiotic stress.

**Figure 4:**
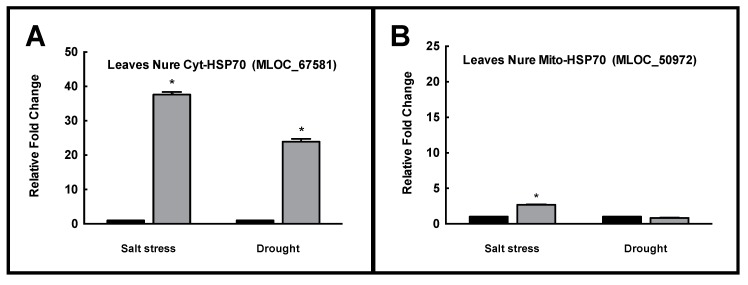
(**A**) The changes in the expression of Cyt-HSP70 (MLOC_67581) (abiotic stress responsive); and (**B**) Mito-HSP70 (MLOC_50972) (biotic stress responsive) in leaves of barley plants cv. Nure collected after 3 days of exposure to salt stress (NaCl 150 mM) and drought (PEG 10%). Asterisks (*) indicate *p* value ≤ 0.001.

As showed in Figure 4, barley plants showed a consistently increased expression of cytosolic HSP70s (MLOC_67581) both upon NaCl (over 37-fold change) and drought (over 23-fold change) compared with the control. 

The mitochondrial HSP70(MLOC_50972) showed a slight 2.5 increase of expression upon salinity while no significant differences were reported upon drought. 

### 2.5. Effects of Abiotic Stress in in Different Barley Genotypes 

**Figure 5:**
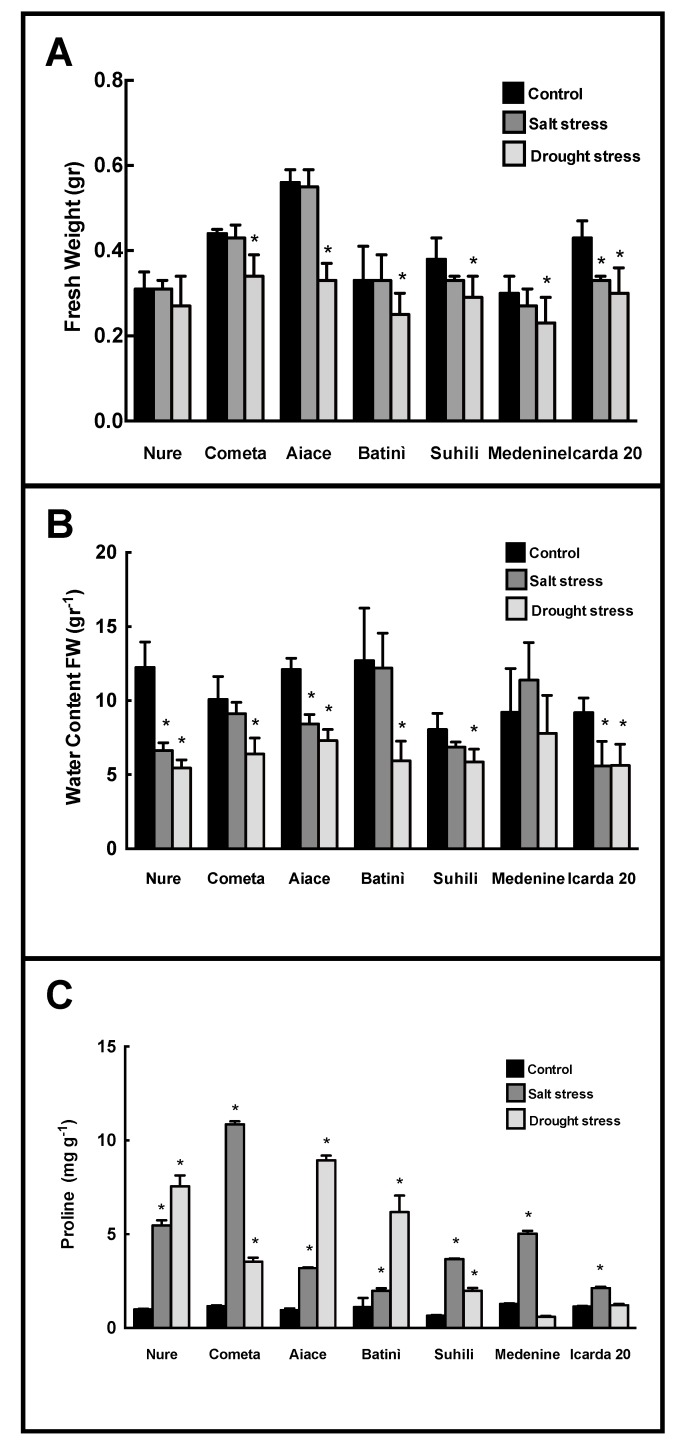
The changes in (**A**) Fresh weight; (**B**) Relative water content (RWC) and (**C**) Proline levels, in leaves of selected barley genotypes in control condition (black bars), Salt stress (NaCl 150 mM) (dark grey bars); and drought (PEG 10%) (light grey bars). The asteriks (*) indicate *p* value ≤ 0.001.

The different barley genotypes and landraces were exposed to salinity and drought. When fresh weight was measured, salt stress did not induce severe changes in all genotypes, except Icarda 20 (−25%). A general and significant decrease was measured upon drought, with Nure (−13%) as the most resistant, and Icarda 20 and Batinì (−32–39%, respectively) as the most susceptible varieties (Figure 5A). 

The relative water content consistently decreased in almost all varieties upon both salinity and drought. Cometa, Batinì, Suhili and Medenine were unaffected by salinity and only Medenine did not exhibit significant changes upon drought (Figure 5B).

These results were evidently counteracted by an increase in Proline. The highest increase was observed in Cometa under salt stress, and Aiace under drought. In Medenine and Icarda 20, the proline increase was among the lowest under salinity, and not significant under drought (Figure 5C). 

Generally, abiotic stress induced severe effects in Italian genotypes, while Northern Africa landraces showed peculiar responses to abiotic stress. Icarda 20 appears less susceptible to treatment. The salt stress response was lower in Batinì, Suihili and Medenine.

### 2.6. HSP70s in Different Barley Genotypes 

The HSP70s isoforms occurrence was further analyzed in barley genotypes from Italy and Northern Africa.

The western blotting analysis indicates that the cytosolic HSP70s display specific occurrence depending on stress treatments (salt or drought). Particularly, all selected genotypes showed an increase of cyt-HSP70 occurrence upon salinity. In contrast, the chl-HSP70 showed no, or reduced, changes in abiotic stress treatments among the various genotypes/landraces. 

**Figure 6:**
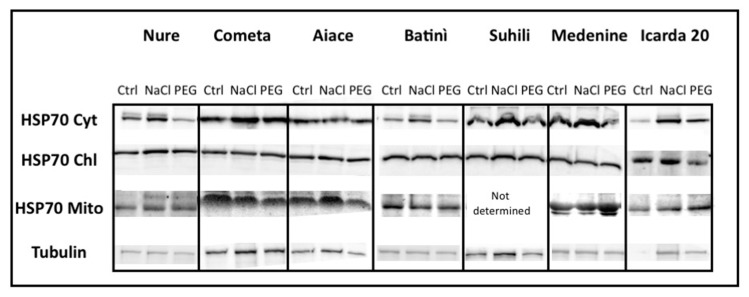
Immunoblotting of leaf extracts from different barley genotypes and landraces plants under control conditions (Ctrl) or exposed to salt stress (NaCl); or drought (PEG) collected after three days. Immunoblotting was performed by using antibodies raised abainst cytosolic (Cy-HSP70); chloroplastic (Chl-HSP70) and mitochondrial (Mito-HSP70). The control blots using anti-tubulin antisera are shown.

The Mito-HSP70 protein occurrence increased in the Icarda 20 genotype under stress, particularly if compared with the Italian genotypes. No appreciable changes were reported in mito-HSP70 protein for Batinì, Suihili and Medenine landraces (Figure 6).

Given these results, Batinì landrace and Icarda 20 genotype were selected for a comparison with the model specie Nure through qRT-PCR analysis in the previously selected HSP70 isoforms encoded by MLOC_67581 (cytosolic, induced by abiotic stress) and MLOC_50972 (mitochondrial, sensitive to pathogen attack). Preliminarily, landraces showed a higher constitutive expression levels of cytosolic MLOC_67581, Batinì 3.4-fold, and Icarda 20 7,7-fold higher with respect to barley Nure (Supplemental Table S2). 

**Figure 7:**
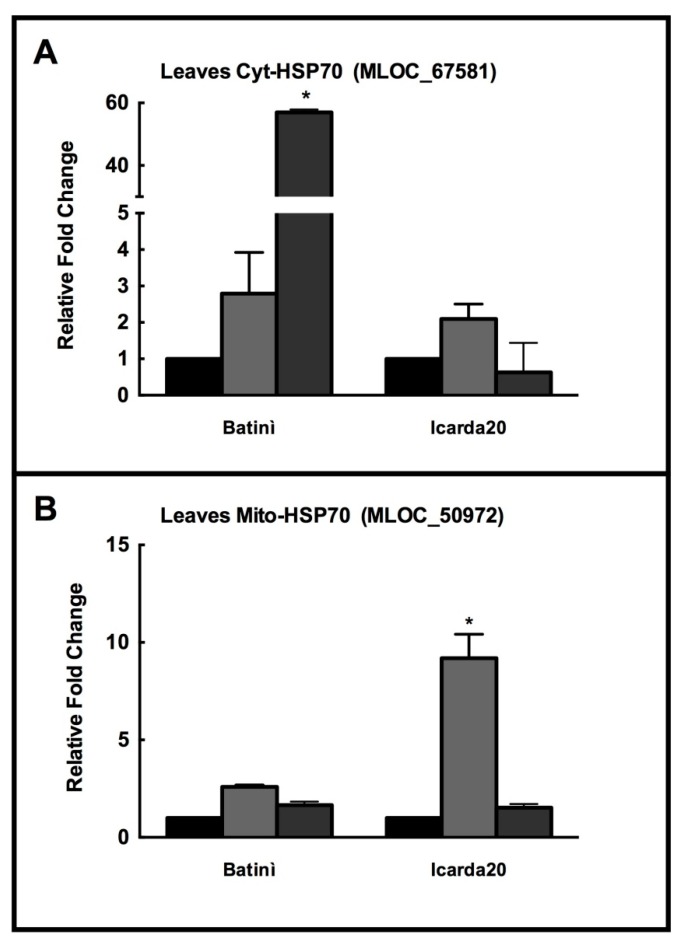
(**A**) The changes in the expression of Cyt-HSP70 (MLOC_67581) (abiotic stress responsive); and (**B**) Mito-HSP70 (MLOC_50972) (biotic stress responsive), in leaves of barley plants cv. Batinì and ICARDA 20 collected after 3 days upon control (Black bars), salt stress (NaCl 150 mM—light grey bars) and drought (PEG 10%—dark grey bars). The asterisks (*) indicate *p* value ≤ 0.001.

As shown in Figure 7A, this higher constitutive level of MLOC_67581 (cytosolic) resulted in a low increase in its expression under salinity, and drought (only in Icarda 20). Batinì landrace showed a strong increase, over 50-fold of this isoform under drought. 

In contrast, mitochondrial HSP70 MLOC_50972 was substantially expressed at very similar levels (Batinì 1.2-fold, and Icarda 20, 0.56-fold with respect to barley Nure) under control conditions (Supplemental Table S2). Batinì showed no change in the expression of the mitochondrial, biotic-stress inducible MLOC_50972 under both salinity and drought. Icarda 20 showed an appreciable increase in the expression of this mitochondrial HSP70 only under salinity (about 10-fold) (Figure 7B).

## 3. Discussion

Barley ranks tenth as the most important produced crop worldwide, with a global cultivation estimated at approximately 143 million tons (FAO stats, 2013; http://faostat3.fao.org/browse/rankings/countries_by_commodity). 

Particularly, 30% of the global barley production is targeted for malting, while 70% to feed use [32]. Traditionally, barley is mainly used as a food crop for human nutrition in the semi-arid countries of Africa (e.g., Morocco, Algeria, and Tunisia), Middle East, the Andean countries of South America and in some Asian counties (e.g., Nepal and Tibet). In European countries such as Germany, France, UK, Denmark and Italy, barley is primarily used for feeding animals [33]. An increased value was given by the managing of the brewing by-products which are feedstock for thermochemical conversion, biogas and ethanol production and other applications [34]. 

In recent years, climate change has reduced the European average production of barley by 3.8% because of the temperature and precipitation changes [35]. This evidence together to the commercial value of barley highlight the need of select new genotypes with improved tolerance to abiotic stress as a strategy to guarantee sustainability [36]. 

This work provided evidence for the contribution of specific HSP70 isoforms in plant responses to different abiotic stresses, namely drought and salinity. 

Recently, different studies described the central role of HSP70s in plants in stress-response conditions [11,12,37]. The drought and salinity conditions used in this work clearly indicated the involvement of selected HSP70 isoforms to counteract the related stresses in barley. 

When barley plants of the cultivar Nure were cultivated in vitro under controlled conditions, and exposed to salinity and drought, a decrease in RWC, and a concomitant increase in proline levels were observed, indicating the effectiveness of the stress imposed. 

The plant response to this stress induced a differential occurrence of distinct HSP70 isoforms— cytosolic HSP70s rapidly increased, particularly upon salinity, but a long-lasting increase was observed upon drought. On the other hand, chloroplastic isoforms remained substantially unaffected under salt stress and increased upon drought conditions, while mitochondrial HSP70s increased under both stresses.

These results pose questions about the identification of distinct HSP70 isoforms induced by stress, and the conditions inducing their specific expression. Therefore, an extensive bioinformatic analysis on barley genome allowed the identification of 20 genes encoding for barley HSP70s, and their localization, the specific tissue expression and the stages of development. Among these, 16 genes are actively expressed in specific tissues and/or specific developmental stages. Similar numbers of HSP70 genes have been recently described in Arabidopsis thaliana [7], pepper [38], rice [6], poplar and *Physchomitrella patens* [39]. 

A quantitative RT-PCR analysis confirmed that the cytosolic isoform strongly increased its expression level upon abiotic stress, while the mitochondrial HSP70 was slightly affected only upon salinity and insensitive to drought. Similar results were showed upon abiotic stresses in other crops as tomato [24,40], pepper [38], rice [41] and others. This identifies key HSP70 genes related to stress tolerance (*Solyc*09g075950, *Solyc*03g117630, *Ca*03g30260 (*Ca*Hsp70-2) and LOC_*Os*08g39140). Intriguingly, analogous roles were identified for the HSP70s to counteract toxic effects of heavy metals in barley upon cadmium stress [37], highlighting the role of this gene family to counteract the effects of unfavorable environments. 

It is therefore clear that the specific response by the HSP70s would greatly ameliorate the adaptation of specific barley cultivars and landraces under abiotic stress conditions.

Barley HSP70s present, as it could be easily assumed, different and multiple cis-acting elements in their promoter regions—cis-elements related to ABA, drought, salinity and other stresses were found in the promoters of the HSP70 genes [13,39]. 

Thus, two isoforms were identified that were supposed to exhibit opposite regulation upon stress. These two HSP70 isoforms are strongly suspected to undergo opposite regulation: The cytosolic HSP70 MLOC_67581, showing the highest number of ABA responsive elements and possibly under abiotic stress control; a mitochondrial isoform, presenting multiple elements involved in fungal/pathogen attack response—HSP70 MLOC_50972—thought to be inducible under pathogen attack. An expression analysis confirmed that in barley Nure, a sensible increase in MLOC_67581 was observed under drought and salinity, while MLOC_50972 was only slightly affected by abiotic stress.

Recently, the detrimental effects of modern breeding and plant domestication were reported to decrease the genomic biodiversity and reduce the abiotic stress tolerance of cultivated crops [42]. The exploitation of landraces and wild relatives is a promising strategy to counteract the genetic erosion [24,42,43,44]. 

In a second set of experiments, this study analyzed the role(s) of HSP70 isoforms in six barley varieties other than Nure: 2 italian genotypes; Aiace and Cometa; one genotype selected by ICARDA (Icarda 20); three barley genotypes and landraces from Tunisia (Suhili, Medenine) and Oman (Batinì). Interestingly, the selected Northern Africa landraces showed peculiar tolerance behavior versus drought and salt stresses. Particularly, the specific protein occurrence and gene expression increases were reported for the HSP70s as well as the proline accumulations. 

Similar opportunities were recently available using barley varieties from northern Asia. Tibetan barley genome (*Hordeum vulgare* L. var. *nudum*), showed a remarkable enlargement in stress-related gene families [45]. Furthermore, Tibetan wild barley (*Hordeum spontaneum* C.) was deeply characterized because of an increased tolerance to salinity and drought obtained by a more efficient sugar and glycine-betaine accumulation, Na^+^/K^+^ ratio regulation, ROS detoxification and others [23,27]. 

These results highlighted the prospective genotypes originated from the semi-arid area of the Mediterranean as a genic source for the improvement of the high-yielding varieties. Among the six varieties investigated, the landrace Batinì showed a different response to salinity, and the improved genotype Icarda 20 resulted as less influenced by both stresses, when compared to their changes in FrWt, RWC and proline levels. These results were substantially confirmed by an immunoblotting analysis on the HSP70s occurrence. 

Following these results, the expression analysis was repeated on the two test HSP70 MLOC_67581 and MLOC_50972 on the landrace Batinì and the selected genotype Icarda 20. Interestingly, both presented an enhanced expression of cytosolic HSP70 MLOC_67581 with respect to Nure. Furthermore, the level of expression did not change upon salinity. Only in the landrace Batinì was a strong enhancement of expression observed under drought conditions. The mitochondrial HSP70 MLOC_50972 did not change, except for a 10-fold increase under salinity in Batinì. These results clearly indicate that the traditional selection of landraces and the modern selection with advanced crossing techniques converge on common molecular traits—in the case here studied, the constitutive overexpression of a stress related cytosolic HsSP70 (MLOC_67581). It is intriguing that in the landrace Batinì the mitochondrial MLOC_50972 is expressed consistently upon salinity, while in the barley genome this promoter does not present cis-acting elements devoted to this stress response. It could be argued that landraces may present changes in both promoter regions of specific stress responding genes. However, the signaling cascade may be changed to adapt to the specific environment. 

These evidences strongly encourage further efforts to identify abiotic stress tolerance alleles of landraces from extreme environments. 

Further studies are necessary to characterize agronomical, physiological and molecular traits of the Northern Africa landraces in different experimental environments. The HSP70 genes from these genotypes could be sequenced and the genomic peculiarities of these genes and of the regulation region can be identified.

## 4. Materials and Methods 

### 4.1. Plant Material and Stress Treatments

The seeds of Italian barley varieties (*Hordeum vulgare*, var. Nure, Cometa and Aiace) were supplied by Centro di ricerca per la genomica e la postgenomica animale e vegetale (CRA-GPG—Fiorenzuola D’Arda—PC, Italy). The seeds of MENA (Middle East North Africa) barley (*Hordeum vulgare*, var. Batinì, Suhili, Medenine and Icarda 20) were supplied by the Laboratory of Genetics and Cereal breeding—INAT, University of Tunis. The genotype’s features were listed in Supplemental Table S3. The seeds were germinated for 7 days in the dark on moistened paper. Then, seedlings were grown in hydroponic solution in darkened plastic bottles at 20 °C, at 60–80% relative humidity, under 16h-light/8h-dark regime, with approximately 180 μmol photons m-2 s-1. The growth medium (modified Hoagland solution) was described in [46]. The solution was continuously aerated.

After 7d in hydroponics, the plants were separated in three groups: The controls were maintained in the standard solution; the salt stress was imposed by adding 150 mM of NaCl to the standard solution; the drought was imposed by the presence of 10% PEG 8000 MW, (Sigma-Aldrich), added to the hydroponic solution. The growth medium was daily controlled for volume and pH and adjusted accordingly. The leaves from Nure genotype were collected at 0 h, 3 h, 6 h, 9 h, 1 day, 3 days and 7 days after the stress induction. The leaves from the other genotypes were collected after 3 days from stress induction.

### 4.2. Growth Variation and Water Content Determination

The changes in the relative water content (RWC) in barley plants exposed to salt and PEG were measured at 0, 3 and 7 days after the stress imposition on 15–20 plants. The plants’ weight was evaluated after hydroponic growth for FW determination. The plants were hydrated for 2–3 h by either floating in a Petri dish in distilled water and weighed to determine the turgid weight (TW). Then, the samples were dried overnight at 70 °C for dried weight (DW) measurements. The plant’s RWC was calculated as follows: RWC % = (FW − DW) / (TW − DW) × 100 [47].

### 4.3. Proline Content

Proline was measured as in [48]. The powdered leaves (250 mg) were suspended in 1.5 mL of 3% sulphosalicylic acid, filtered through a glass-fiber filter (Macherey-Nagel, Ø 55 mm, Germany). Further, 1ml of glacial acetic acid and 1 mL ninhydrin reagent (2.5 g ninhydrin/100 mL of a 6:3:1 solution of glacial acetic acid, deionized water and 85% orto-phosphoric acid) were added to the filtrate (1 mL). After 1 h at 100 °C, the optical density was read at 546 nm (Cary 60 spectrophotometer—Agilent Technologies, Santa Clara, CA, USA).

### 4.4. Western Blotting

In immunoblottings, the proteins were extracted as previously described and separated by SDS-PAGE [49]. Then, the polypeptides were transferred onto a Hybond nitrocellulose membrane (GE Healthcare, Chicago, IL, USA). The filter was incubated with primary antibodies (Agrisera) versus the HSP70s (Cytosolic, Chloroplastic and mitochondrial) and tubulin. After incubation of the membrane with secondary antibodies, the cross-reacting polypeptides were identified by enhanced chemioluminescence (WesternBright^TM^ Quantum kit—Advansta, San Josè, CA, USA). The images were acquired by BioRad Chemidoc system (Bio-Rad, Hercules CA, USA).

### 4.5. RNA Extraction and qRT-PCR

The RNA extraction was made from the leaves (100 mg) using Bio-Rad Aurum^TM^ Total RNA Mini Kit. The cDNA syntheses were done using the ThermoScript RT-PCR System. The RNA amount was measured by NanoDrop ND-1000 spectrophotometer. The gene expression analysis was carried out by qRT-PCR. Triplicate quantitative assays were made by using Applied Biosystems™ 7500 Real-Time PCR System and Platinum SYBR Green qPCR SuperMix (Life Technologies, Carlsbad, CA, USA). The leaf samples of the control plants were used as calibrators and α-tubulin served as an endogenous reference gene. The quantization of the gene expression was carried out using the 2^−ΔΔCt^ method as in [50]. the mRNA amount was calculated in each sample, relative to the calibrator sample for the same gene. A list of primers is provided in Supplemental Table S4. 

### 4.6. Bioinformatics Analysis

The sequences of barley HSP70s were found using barley genome at https://ics.hutton.ac.uk/morexGenes/. The sequences from different species were from the TAIR database (https://www.arabidopsis.org) and the Ensamble plants database. The alignments and phylogenetic analyses were made by the software MEGA 6.0 [51]. The alignments were obtained by MUSCLE algorithm. The phylogenetic tree was designed by using the maximum likelihood method with the JTT substitution model. The test of phylogeny was performed using a bootstrap method (bootstrap replication = 100). The conserved motif analysis was performed by MEMESuite4.11.1 server 5 [52]. The promoter analyses were performed at Plant CARE server suites using regions of 1000 bp upstream from the start codons of each HSP70 gene [53]. An *Arabidopsis thaliana* orthologs co-expression analysis was carried out by ATTED-II versus 8.0 (http://atted.jp). The degree of co-expression was estimated as mutual rank [54]. The expression analysis in different tissues was retrieved using the database at https://ics.hutton.ac.uk/morexGenes/.

### 4.7. Statistics

The experiments were made in at least three replicates. The values were expressed as the mean ± standard error and statistical through the Student’s *t*-test (*p* ≤ 0.05). The ANOVA analysis was used to compute the statistical significance of differences between the controls and the stressed groups and between different genotypes (ANOVA corresponds to α = 0.05). The Tukey–Kramer post-hoc test was used to evaluate differences between the means.

## Figures and Tables

**Table 1:** List of identified barley HSP70s, their localization and pfam identified domains.

Locus	Localization	Proposed Nomenclature	Pfam Domains IDs
MLOC_12446	Cytoplasm	HvHSP70	PF00012.20; PF06723.13
MLOC_14228	Cytoplasm	HvHSP70	PF00012.20; PF06723.13
MLOC_15242	Mitochondrial	HvMithHSP70	PF00012.20; PF06723.13
MLOC_2467	Cytoplasm	HvHSP70	PF00012.20; PF06723.13
MLOC_26505	Cytoplasm	HvHSP70	PF00012.20; PF06723.13
MLOC_37101	Chloroplast	HvCHPHSP70	PF00012.20; PF06723.13
MLOC_4447	Cytoplasm	HvHSP70	PF00012.20; PF06723.13
MLOC_45046	Cytoplasm	HvHSP70	PF00012.20; PF06723.13
MLOC_50972	Mitochondrial	HvMithHSP70	PF00012.20; PF06723.13; PF02782.16
MLOC_53941	Cytoplasm	HvHSP70	PF00012.20; PF06723.13
MLOC_55086	Chloroplast	HvCHPHSP70	PF00012.20; PF06723.13
MLOC_55096	Cytoplasm	HvHSP70	PF00685.27
MLOC_55999	Cytoplasm	HvBIP	PF00012.20; PF06723.13
MLOC_61727	Mitochondrial	HvMithHSP70	PF00012.20; PF06723.13
MLOC_65512	Cytoplasm	HvHSP70	PF00012.20; PF06723.13
MLOC_67581	Cytoplasm	HvHSP70	PF00012.20
MLOC_72334	Cytoplasm	HvHSP70	PF00012.20; PF06723.13
MLOC_76167	Cytoplasm	HvHSP70	PF00012.20
MLOC_77827	Cytoplasm	HvBIP	PF00012.20; PF06723.13
MLOC_78867	Cytoplasm	HvHSP70	PF00012.20; PF06723.13

**Table 2:** The expression analysis of barley’s HSP70 genes obtained at https://ics.hutton.ac.uk/morexGenes in different development stages and tissues: Embryogenesis, seedling shoots and roots, inflorescences (young and development), development tillers and development grains (5 and 15 days post anthesis DPA). The data are expressed as FPKM normalized counts of three different replicates. The colors represent the expression value from lower values (0—red) from higher values (green).

	Expression data (FPKM)							
	4 Days Embryo	Seedling Shoots	Young Inflorescences	Developing Inflorescences	Seedling Roots	Developing Tillers	Developing Grains (5 DPA)	Developing Grains (15 DPA)
MLOC_12446	928.19	674.36	1584.58	1909.81	1181.38	354.58	524.31	216.92
MLOC_14228	39.44	202.24	1.21	3.92	418.49	4.72	72.14	220.23
MLOC_15242	33.28	33.49	32.72	28.73	38.93	1.82	38.89	6.71
MLOC_2467	224.4	139.3	166.2	182.7	203.6	308.2	193.2	123.5
MLOC_26505	45.00	67.59	50.43	68.15	67.48	11.90	94.58	60.19
MLOC_37101	135.86	237.33	139.46	98.02	60.39	71.76	116.04	45.66
MLOC_4447	0.04	13.67	0.02	2.03	32.01	0.27	0.38	43.40
MLOC_45046	101.50	336.15	3.47	2.63	226.17	210.66	77.81	20.03
MLOC_50972	171.19	103.86	196.05	153.72	109.15	39.96	170.41	44.16
MLOC_53941	162.85	134.29	322.87	549.16	175.32	92.78	302.49	79.18
MLOC_55086	51.30	125.24	45.13	46.13	69.61	36.50	118.59	86.64
MLOC_55096	1.68	0.13	0.00	0.00	2.58	0.53	0.04	0.00
MLOC_55999	267.44	166.85	126.02	118.81	259.95	64.44	720.53	146.05
MLOC_61727	3.53	11.46	0.92	12.78	23.20	0.23	13.54	26.91
MLOC_65512	0.00	0.03	0.00	0.01	1.62	0.00	0.00	0.01
MLOC_67581	0.80	6.53	0.40	0.58	17.17	0.01	2.02	44.56
MLOC_72334	2.86	16.57	0.08	0.16	54.85	0.05	5.23	6.86
MLOC_76167	36.80	27.78	52.17	54.60	27.87	46.08	44.69	13.62
MLOC_77827	0.47	0.18	0.07	0.01	0.65	0.37	0.29	0.24
MLOC_78867	336.38	242.84	726.65	1088.08	384.78	169.08	511.81	164.32

**Table 3:** The regulatory cis-acting elements of the barley HSP70s promoters (1500 pb upstream region). The legend for motifs: ABRE and motif IIB(abscisic acid response); ARE (anaerobic induction); AUXRR-COR (auxin response); GCTCA & TGAGC (Me-Jasmonate—response); Box W1 & EIRE (Elicitor responsive elements); ERE (ethylene-responsive element); GARE & P-BOX (gibberellin-responsive element); GCN4 & Skn-1_motif (regulatory element required for endosperm expression); HSE (heat stress response); MBS (MYB binding site involved in drought response); LTR (in low-temperature response); p-BOX (gibberellin-responsive element); Ry elements (seed-specific regulation); TATC (gibberellin-response); TC-rich repeats (defense and stress response); TCA (salicylic acid response); TGA (auxin- response).

	Cis-Actig Elements in Promoter Region																						
	ABRE	ARE	AUXRR-Core	BOX W1	CCAAT	CGTCA	ERE	EIRE	GARE	GCN4	HSE	Light	LTR	P-box	MBS	Motif IIB	Ry	SKN-1	TATC	TC-rich	TCA	TGA	TGAGC
MLOC_12446	0	1	1	0	1	0	2	0	0	1	2	8	0	0	1	0	0	3	1	3	0	0	0
MLOC_14228	1	1	0	0	0	0	0	0	1	0	0	4	0	1	0	0	0	1	0	1	0	0	0
MLOC_15242	*No available data*																						
MLOC_2467	0	0	2	0	1	4	0	1	3	0	1	18	4	0	2	0	0	1	0	2	0	1	4
MLOC_26505	2	1	0	1	0	2	0	0	0	1	1	7	1	0	0	1	0	2	0	1	0	0	2
MLOC_37101	*No available data*																						
MLOC_4447	0	1	0	0	0	0	0	0	1	2	2	14	1	0	0	0	0	1	0	3	2	0	0
MLOC_45046	2	2	0	1	0	1	1	0	1	0	1	17	2	0	2	0	1	2	0	0	0	0	1
MLOC_50972	0	0	0	3	1	3	0	0	1	0	1	20	1	0	4	1	0	5	0	1	0	0	3
MLOC_53941	1	1	0	0	0	1	0	0	3	0	0	10	1	1	3	0	1	5	0	1	1	0	1
MLOC_55086	*No available data*																						
MLOC_55096	2	2	0	0	0	1	0	0	3	1	1	27	0	0	14	0	1	7	0	0	0	0	1
MLOC_55999	3	0	0	2	0	2	0	0	1	0	2	17	0	0	0	0	0	2	0	1	0	0	2
MLOC_61727	0	0	0	1	0	4	0	0	0	0	1	18	1	0	0	0	0	3	0	0	0	1	4
MLOC_65512	0	2	0	1	0	1	0	0	0	1	1	11	1	0	1	0	0	4	1	1	1	1	1
MLOC_67581	4	2	0	1	2	2	0	1	1	0	0	19	2	0	1	0	2	8	0	0	1	0	2
MLOC_72334	0	0	0	3	2	2	0	0	0	1	0	15	0	1	1	0	0	4	0	2	1	1	2
MLOC_76167	*No available data*																						
MLOC_77827	*No available data*																						
MLOC_78867	3	0	0	0	0	2	0	0	0	0	1	15	0	0	2	0	0	3	0	0	1	2	2

## Supplementary Materials

The following are available online at https://www.mdpi.com/2223-7747/8/8/248/s1, Table S1: Co-expression analysis of Arabidopsis HSP70S orthologous, obtained using the ATTED-II database, Table S2: Expression rate of MLOC_67581 and MLOC_50972 by qRT-PCR in barley Batinì and Icarda 20 vs. Barley Nure, Table S3: List of selected genotypes of barley (*Hordeum vulgare*) utilized in this study, Table S4: List of primers used for qRT-PCR analysis.
