# Supplementary material for: Different Roles of Heat Shock Proteins (70 kDa) During Abiotic Stresses in Barley (Hordeum vulgare) Genotypes
Source: Plants (Basel). 2019 Jul 26;8(8):248. doi: 10.3390/plants8080248 (PMC6724185; doi:10.3390/plants8080248)
Supplement: Supplementary file 1 [file plants-08-00248-s001.pdf]

**LANDI et al., 2019 - HSP70 Barley upon abiotic stresses**

**SUPPLEMENTARY FILES**

**Supplementary Table S1**

Co-expression analysis of Arabidopsis HSP70S orthologous, obtained using the ATTED-II database. The first 15 co-expressed genes were selected for the table.

Co-expression degree was estimated as Mutual Rank, as described in **[**54].

| **At5g02500** | **At5g02490** | **At3g09440** | **At3g12580** | **At1g16030** |
|---|---|---|---|---|
| DNAJ homologue 3 | Heat shock protein 70 | heat shock protein 81-2 | heat shock protein 90.1 | HSP20-like chaperones |
| heat shock protein 81-2 | DNAJ homologue 2 | PPPDE putative thiol peptidase | DNAJ heat shock family protein | BCL-2-associated athanogene 6 |
| Heat shock protein 70 | heat shock protein 81-2 | DNAJ homologue 3 | heat shock protein 101 | HSP20-like chaperones superfamily protein |
| heat shock protein 60 | heat shock protein 90.1 | Heat shock protein 70 | stress-inducible protein, putative | heat shock protein 17.4 |
| DNAJ homologue 2 | DNAJ homologue 3 | DNAJ homologue 2 | ATPase, AAA-type, CDC48 protein | heat shock protein 17.6A |
| ABC transporter family protein | mitochondrial HSO70 2 | serine/threonine phosphatase 2A | HSP20-like chaperones | heat-shock protein 70T-2 |
| Heat shock protein 70 | Fes1A | heat shock cognate protein 70-1 | multiprotein bridging factor 1C | 17.6 kDa class II heat shock protein |
| S-adenosyl-L-homocysteine hydrolase | heat shock protein 70 | mitochondrial HSO70 2 | FKBP-type peptidyl-prolyl cis-trans isomerase | mitochondrial small heat shock protein 23.6 |
| mitochondrial HSO70 2 | PPPDE putative thiol peptidase | rotamase FKBP 1 | Fes1A | HSP20-like chaperones |
| Aha1 domain-containing protein | Aha1 domain-containing protein | Chaperone DnaJ-domain | SERINE-ARGININE PROTEIN 30 | heat shock protein 21 |
| MIF4G domain-containing protein | heat shock cognate protein 70-1 | stress-inducible protein, putative | PPPDE putative thiol peptidase family protein | At3g07150 |
| Ribosomal protein S5 | pyridoxine biosynthesis 1.2 | DNAJ heat shock family protein | heat shock transcription factor A2 | P-loop nucleoside triphosphate hydrolases |
| DEAD box RNA helicase (PRH75) | DNAJ heat shock | Fes1A | RNA-binding (RRM/RBD/RNP motifs) | heat shock protein 101 |
| HSP20-like chaperones | Argonaute family protein | heat shock protein 70 | TIN1 | HSP20-like chaperones superfamily protein |
| pyridoxine biosynthesis 1.2 | non-ATPase subunit 9 | Aha1 domain-containing protein | Cytochrome bd ubiquinol oxidase | Chaperone DnaJ-domain superfamily protein |
| **At4g24280** | **At5g49910** | **At2g32120** | **At4g37910** | **At5g09590** |
| chaperonin-60alpha | TCP-1/cpn60 chaperonin | heat shock protein 101 | heat shock protein 60-2 | rotamase FKBP 1 |
| TCP-1/cpn60 chaperonin | At2g43630 | HSP20-like chaperones | nucleoside triphosphate hydrolases | ankyrin repeat family protein |
| Chaperone protein htpG | DNAJ heat shock | HSP20-like chaperones | hydroxyproline-rich glycoprotein | heat shock protein 81-2 |
| chaperonin 60 beta | chaperonin-60alpha | heat shock protein 70B | elongation factor family protein | HEAT SHOCK PROTEIN 89.1 |
| translocon at membrane of chloroplasts 11 | chloroplast heat shock protein 70-1 | gravitropism-deficient-yellow-green-like 3 | lon protease 1 | heat shock protein 60 |
| chloroplast heat shock protein 70-2 | Chaperone protein htpG | HSP20-like chaperones | Haloacid dehalogenase-like hydrolase (HAD) | HSP20-like chaperones superfamily protein |
| RNI-like superfamily protein | heat shock protein 81-2 | heat shock protein 17.6A | GTP binding Elongation factor | Heat shock protein 70 (Hsp 70) family protein |
| FTSH protease 12 | glycyl-tRNA synthetase | stress-inducible protein, putative | heat shock protein 60 | casein lytic proteinase B4 |

| | | | | |
|---|---|---|---|---|
| Class II aaRS and biotin synthetases | Heat shock protein 70 | heat shock protein 17.4 | lysyl-tRNA synthetase 1 | stress-inducible protein, putative |
| Octicosapeptide/Phox/Bem1p (PB1) | SWIB/MDM2 domain;Plus-3;GYF | At4g23493 | Ribosome recycling factor | DNAJ homologue 3 |
| RNA-binding CRS1 / YhbY | rotamase FKBP 1 | mitochondrial small heat shock protein 23.6 | Glutathione S-transferase | Phosphoglycerate mutase |
| plastid transcriptionally active 12 | arogenate dehydratase 1 | 17.6 kDa class II heat shock protein | prohibitin 2 | DNAJ homologue 2 |
| presequence protease 1 | zinc finger (Ran-binding) | HSP20-like chaperones | translocon at outer membrane of chloroplasts 6 | Glutathione S-transferase family protein |
| glycine-tRNA ligases | plastidial pyruvate kinase 3 | galactinol synthase 1 | voltage dependent anion channel 3 | RNA-binding (RRM/RBD/RNP motifs) |
| elongation factor Ts family protein | aspartate kinase-homoserine dehydrogenase ii | heat shock protein 70 | heat shock protein 60-3° | SGS domain-containing protein |
| **At1g09080** | **At1g11660** | **At4g16660** | **At1g56410** | |
| DNAJ heat shock N-terminal | Chaperone protein htpG | Chaperone protein htpG family protein | At4g07720 | |
| Preprotein translocase Sec | DNAJ heat shock family protein | DNAJ heat shock family protein | At4g16090 | |
| cysteine-rich RLK | glutamine-fructose-6-phosphate transaminasi | glutamine-fructose-6-phosphate transaminase | UDP-Glycosyltransferase superfamily | |
| UDP-Glycosyltransferase | calnexin 1 | calnexin 1 | Cysteine proteinases superfamily protein | |
| endoplasmic reticulum oxidoreductins 1 | PDI-like 1-2 | PDI-like 1-2 | F-box domains protein | |
| TIN1 | Vps51/Vps67 | At4g29520 | At5g38400 | |
| RECEPTOR-like protein kinase n36 | Coatomer, beta' subunit | Coatomer, beta' subunit | zinc ion binding;nucleic acid binding | |
| receptor like protein 41 | calreticulin 1° | calreticulin 1a | transposable element gene | |
| nucleoside triphosphate hydrolases | calreticulin 1b | calreticulin 1b | transposable element gene | |
| wall associated kinase 3 | DnaJ / Sec63 Brl domains-containing protein | DnaJ / Sec63 Brl domains-containing protein | transposable element gene | |
| UDP-galactose transporter 1 | PDI-like 1-1 | PDI-like 1-1 | glycine-rich protein | |
| receptor like protein 23 | UDP-galactose transporter 3 | UDP-galactose transporter 3 | At3g50250 | |
| 2-oxoglutarate (2OG) oxygenase | stromal cell-derived factor 2-like precursor | stromal cell-derived factor 2-like protein precursor | Galactose oxidase/kelch repeat superfamily protein | |
| PDI-like 1-2 | PDI-like 2-2 | PDI-like 2-2 | At2g11010 | |
| cysteine-rich RLK 37 | UDP-galactose transporter 1 | UDP-galactose transporter 1 | Plant EC metallothionein-like protein, family 15 | |

[54] Aoki Y.; Okamura Y.;, Tadaka S.; Kinoshita K.; Obayashi T. ATTED-II in 2016: A plant co-expression database towards lineage-specific co-expression. *Plant Cell Physiol* **2016** *57*, e5(1–9). doi: 10.1093/pcp/pcv165

**Supplementary Table S2** – Expression rate of selected loci MLOC_67581 and MLOC_50972 measured by qRT-PCR analysis in barley Batinì and Icarda 20 vs Barley Nure under control conditions. The expression rate in barley Nure has been arbitrarily assigned as 1, and expression ratios with respect to other varieties calculated accordingly.

| LOCUS | Name | Nure (reference) | Batinì (fold-change) | Icarda 20 (fold-change) |
|---|---|---|---|---|
| MLOC_67581 | HSP70 Cyt | 1 | **3.43** | **7.66** |
| MLOC_50972 | HSP70 Mito | 1 | **1.26** | **0.56** |

**Supplementary Table S3 –** List of selected genotypes of barley (*Hordeum vulgare*) utilized in this study.

| Barley variety | Crossing | Variety | Production | Landrace/genotype from |
|---|---|---|---|---|
| Nure | (FIOR+40+x+ALPHA2+) x BARAKA | Two-Row | Seeds + Forage | Italy |
| Aiace | FO+1078+x+FO+1638 | Two-Row | Seeds + Forage | Italy |
| Cometa | PO202.169x+F2FO3356 (Amillis x+Fior2377) | Two-Row | Forage | Italy |
| Batinì | Traditional landrace | Six-Row | Seeds | Oman |
| Suihili | Traditional landrace | Six-Row | Seeds + Forage | Tunisia |
| Medenine | Traditional landrace | Six-Row | Seeds + Forage | ICARDA/Tunisia |
| Icarda20 | Improved | Six-Row | Seeds + Forage | ICARDA |

**Supplementary Table S4 –** List of primers used for qRT-PCR analysis.

| For/Rev | Sequences | Description |
|---|---|---|
| Forward | CTCCATGATGGCCAAGTGTGA | Alfa-tub |
| Reverse | ATGTCGCTTGGTCTTGATGGT | |
| Forward | CCTTGGTTGCTCCTCCAGTA | MLOC_67581 |
| Reverse | TGCTGAAAAACACTCGCAGC | |
| Forward | AGCAGCATCAGCCGTGATAA | MLOC_50972 |
| Reverse | TCTGCAAAGGTTGCGTGAAG | |
| Forward | CTACCAGCAGCAGGGATCTG | MLOC_55086 |
| Reverse | CACCATCGCCTGTCTTCTCA | |
| Forward | TAAGGCTTGCCCTCCTTGTT | MLOC_55999 |
| Reverse | ACGTCAAGCGTCTCATTGGA | |
| Forward | CCTCAGCTGCAAACTGCTTC | MLOC_53941 |
| Reverse | CTGTGGCCATTCAAGGTTGT | |
